# Molecular Evolution of Dengue Virus 3 in Senegal between 2009 and 2022: Dispersal Patterns and Implications for Prevention and Therapeutic Countermeasures

**DOI:** 10.3390/vaccines11101537

**Published:** 2023-09-28

**Authors:** Idrissa Dieng, Diamilatou Balde, Cheikh Talla, Diogop Camara, Mamadou Aliou Barry, Samba Niang Sagne, Khadim Gueye, Cheikh Abdou Khadre Mbacké Dia, Babacar Souleymane Sambe, Gamou Fall, Amadou Alpha Sall, Ousmane Faye, Cheikh Loucoubar, Oumar Faye

**Affiliations:** 1 Arboviruses and Haemorrhagic Fever Viruses Unit, Virology Department, Institut Pasteur de Dakar, Dakar 220, Senegal; djamsbalde@gmail.com (D.B.); diogop.camara@pasteur.sn (D.C.); gamou.fall@pasteur.sn (G.F.); amadou.sall@pasteur.sn (A.A.S.); ousmane.faye@pasteur.sn (O.F.); oumar.faye@pasteur.sn (O.F.); 2 Epidemiology, Clinical Research and Data Science Department, Institut Pasteur de Dakar, Dakar 220, Senegal; cheikh.talla@pasteur.sn (C.T.); aliou.barry@pasteur.sn (M.A.B.); sambaniang.sagne@pasteur.sn (S.N.S.); cheikh.loucoubar@pasteur.sn (C.L.); 3 EMBL’s European Bioinformatics Institute, Hinxton, Cambridge CB10 1SD, UK; gueyekgy@gmail.com; 4 Department of Animal Biology, Faculty of Science et Technics, Université Cheikh Anta Diop de Dakar (UCAD), BP 5005 Fann, Dakar, Senegal; imam901@gmail.com (C.A.K.M.D.); babacarsouleymane.sambe@pasteur.sn (B.S.S.)

**Keywords:** DENV−3, molecular evolution, Senegal, amino acid changes, vaccine strain, human mAB

## Abstract

Dengue fever is the most prevalent arboviral disease worldwide. Dengue virus (DENV), the etiological agent, is known to have been circulating in Senegal since 1970, though for a long time, virus epidemiology was restricted to the circulation of sylvatic DENV−2 in south-eastern Senegal (the Kedougou region). In 2009 a major shift was noticed with the first urban epidemic, which occurred in the Dakar region and was caused by DENV−3. Following the notification by Senegal, many other West African countries reported DENV−3 epidemics. Despite these notifications, there are scarce studies and data about the genetic diversity and molecular evolution of DENV−3 in West Africa. Using nanopore sequencing, phylogenetic, and phylogeographic approaches on historic strains and 36 newly sequenced strains, we studied the molecular evolution of DENV−3 in Senegal between 2009 and 2022. We then assessed the impact of the observed genetic diversity on the efficacy of preventive countermeasures and vaccination by mapping amino acid changes against vaccine strains. The results showed that the DENV−3 strains circulating in Senegal belong to genotype III, similarly to strains from other West African countries, while belonging to different clades. Phylogeographic analysis based on nearly complete genomes revealed three independent introduction events from Asia and Burkina Faso. Comparison of the amino acids in the CprM-E regions of genomes from the Senegalese strains against the vaccine strains revealed the presence of 22 substitutions (7 within the PrM and 15 within the E gene) when compared to CYD-3, while 23 changes were observed when compared to TV003 (6 within the PrM and 17 within the E gene). Within the E gene, most of the changes compared to the vaccine strains were located in the ED-III domain, which is known to be crucial in neutralizing antibody production. Altogether, these data give up-to-date insight into DENV−3 genomic evolution in Senegal which needs to be taken into account in future vaccination strategies. Additionally, they highlight the importance of the genomic epidemiology of emerging pathogens in Africa and call for the implementation of a pan-African network for genomic surveillance of dengue virus.

## 1. Introduction

Dengue fever (DF) is caused by dengue virus (DENV), a mosquito-borne virus belonging to the genus Flavivirus in the family of Flaviviridae [1]. DENV is a single-stranded, positive-sense RNA virus coding for seven non-structural proteins and three structural proteins [2]. DENV exists in four genetically and antigenically distinct forms named serotypes DENV−1, DENV−2, DENV−3, and DENV−4 [3]. According to WHO estimates, 390 million infections occur each year, of which 96 million are clinically apparent [1]. DENV is considered to be the most prevalent and rapidly spreading arboviral infection worldwide, with tropical and subtropical regions most affected [1]. DF is thought to be rare in Africa [4]. In contrast, according to recent estimates, 16% of all clinically apparent DENV infections occur in Africa [5], highlighting likely underreporting. Virus occurrence underestimation in the African continent is linked to the paucity of effective syndromic surveillance systems, low awareness of healthcare givers, other prevalent malaria-like illnesses which lead to misdiagnoses, and the lack of diagnostic tools [6]. Previous studies report that most of the epidemics in the African continent were linked to DENV−2, followed by DENV−1 [6]. However, more recently, DENV−3 has become endemic [7,8] and has been found to be actively circulating in West Africa since 2006 [9].

In Senegal, as in Africa, all DENV serotypes have been described [10] with multifocal and multiserotype circulation since 2017 [11]. According to data from the WHO collaborating center (WHOCC) for arboviruses and hemorrhagic fever viruses, most confirmed dengue cases detected in Senegal through the 4S network surveillance program between 2018 and 2022 were DENV−3 (unpublished data). Additionally, epidemics caused by DENV−3 were reported in Agnam Civol, which is located in north-eastern Senegal, in 2021 and 2022 (manuscript in preparation), as well as in the Touba area in 2018, with 293 confirmed cases [12], and it was the dominant serotype during a DF outbreak in Thies region in 2018 that was marked by the cocirculation of DENV 1–3 [13]. Despite recurrent reports of DENV−3 in Senegal, there are limited studies assessing the genetic diversity of circulating strains in the country.

Major efforts in DENV research are currently focused on designing, producing, and licensing a tetravalent vaccine [14]. It is important, therefore, to understand the role of DENV genetic variability on vaccine efficacy [15]. Deep knowledge of virus genetic diversity and dispersal patterns in many African countries is hampered by the absence of genomic data [16]. DENV sequence data from Africa represent less than 1% of those deposited in Genbank. Improved viral genomic surveillance can assist in better understanding viral transmission dynamics in Africa and guide vaccination policies [17].

Herein, leveraging nanopore sequencing technology, we study the molecular evolution of Senegalese DENV−3 strains between 2009 and 2022. We then provide insights into the in silico impact of the viral genetic make-up on the efficacy of preventive and therapeutic countermeasures.

## 2. Materials and Methods

### 2.1. Sample Selection

**Figure 1:**
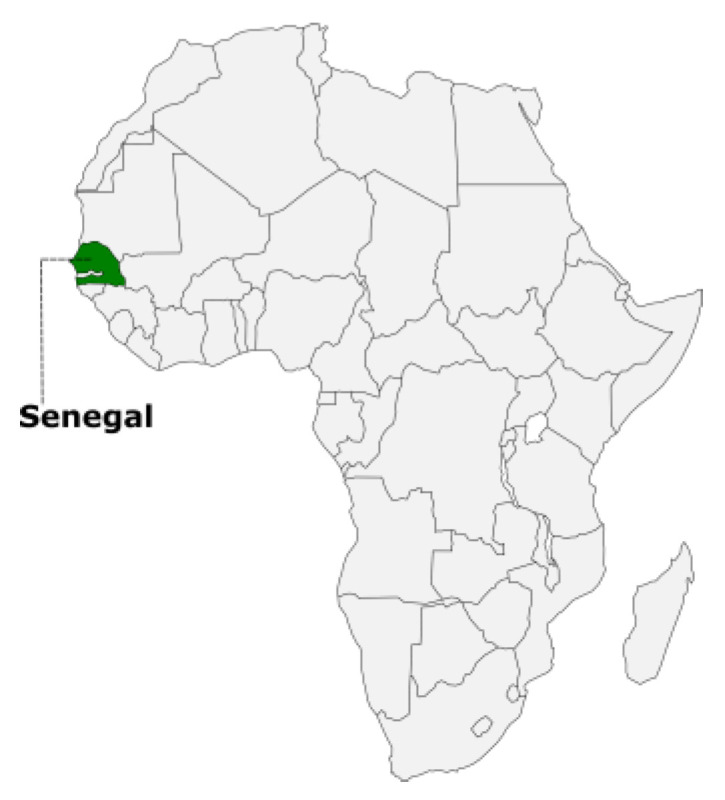
Map showing the geographical location of Senegal.

Samples were collected from patients with suspected arboviral infection as part of the ongoing syndromic surveillance of fever performed by the Institut Pasteur of Dakar (IPD), in collaboration with the Senegalese Ministry of Health [11], around Senegal (Figure 1) between January 2019 and March 2022. For each eligible patient who met the previously described case definition for DENV [18], a 5 mL blood sample was collected using venipuncture into a Vacutainer tube. Collected samples were transiently conserved at +4 at the sentinel site and shipped with their corresponding demographic/epidemiological forms to IPD on a weekly basis. At IPD, samples were tested for 7 medically important arboviruses, including dengue virus, Zika virus, Chikungunya virus, Rift Valley fever virus, West Nile virus, yellow fever virus, and Crimean–Congo virus.

### 2.2. RNA Extraction

At IPD, shipped blood samples were de-identified and a unique identifier was provided to each venous blood tube. Blood samples were centrifuged at 2000 rpm for 5 min to separate serum from blood coat and sera were stored in cryotubes. A total of 140 µL was used as input for RNA extraction using Qiagen Viral RNA mini kit (Qiagen, Hilden, Germany) according to the manufacturer’s recommendations. Extracted RNA was eluted in a final volume of 60 µL and used immediately or stored at −80 before further use.

### 2.3. Dengue Virus Detection and Serotyping with qRT-PCR

Extracted RNA of selected suspected DENV samples was subjected to molecular testing to assess viral genome presence. The 3′ untranslated region (3′UTR) of the DENV was amplified through quantitative reverse transcription polymerase chain reaction (qRT-PCR). The primers employed for amplification were previously described by Wagner and colleagues [19]. All primers were synthetized at TIBMolbiol (Berlin, Germany) at a concentration of 5 nM.

The qRT-PCR was executed using the AgPath-ID One-step RT-PCR kit on a Quantstudio cycler (Thermo Fisher Scientific, Waltham, MA, USA). Samples with a cycle threshold (Ct) value below 35 were categorized as DENV RNA-positive. To identify the specific serotype of DENV in the positive samples identified through the pan-DENV RT-qPCR, real-time molecular serotyping was performed using the same RNA extracts. This serotyping was conducted using the TIBMolbiol Modular Dx Dengue Typing Kit (TIBMolbiol, Berlin, Germany) (Cat-No. 40-0700-24), a commercially available kit, following a protocol published previously [20]. This kit allows for the simultaneous detection of all four DENV serotypes in a single reaction, utilizing only 5 µL of input RNA.

### 2.4. cDNA Synthesis and Tilling PCR Amplification

RNA from confirmed DENV−3-positive samples were used as a template for first-stranded cDNA synthesis using the Lunascript RT Supermix kit (New England Biolab, County Road, Ipswich, MA, USA) according to the manufacturer’s recommendations. An amplicon-based approach was then performed by using a multiplex PCR primer scheme, similar to the one previously described by Dieng and colleagues [21], that was designed to amplify the entire coding region of DENV−3 in two pools. All PCR amplification reactions were performed using Q5 high-fidelity 2X master mix (New England Biolabs). Product average size (~900 bp) was checked using a 1.5% agarose gel.

### 2.5. Sequencing and Genome Assembly

Amplicons from DENV−3-positive samples were subsequently subjected to sequencing using nanopore technology.

In summary, the amplicons were purified using Ampure beads in a 1:1 ratio. For sequencing, these purified amplicons were tagged using the Rapid Barcoding Kit 96 (SQK-RBK110.96, Nanopore technologies, Oxford, UK), following the manufacturer’s instructions. Additionally, a blank control sample with water was included. The resulting libraries were quantified, normalized, pooled together, and loaded onto R9.4.1 flow cells (FLO-MIN106D, Nanopore technologies, Oxford, UK) on the Oxford Nanopore MinION platform, where they underwent sequencing for at least 24 h. The sequencing reads were processed using Guppy software version 4.5.4, which is available at https://community.nanoporetech.com. These reads were merged into a single Fastq file using a bash command line, and consensus sequences were generated using the genome detective online tool, which can be accessed at https://www.genomedetective.com/ (accessed intermittently between 18 September 2022).

To determine the genotype of the newly assembled DENV−3, the genome detective dengue typing tool was employed, and it can be accessed at https://www.genomedetective.com/app/typingtool/dengue/ (accessed on 11 September 2022).

### 2.6. Dataset Construction

The nearly complete genomes acquired during this study (*n* = 36) were merged with a representative subset of previously collected global full-length DENV−3 sequences. Information detailing the location and year of isolation was gathered from the National Center of Biotechnology Information (NCBI) in GenBank format and subsequently transformed into FASTA format. This compilation resulted in dataset 1, which included sequences available up to April 2022

To determine genotypes and lineages associated with Senegalese DENV−3 sequences covering the study period, we constructed a dataset containing Senegalese sequences in addition to all described genotypes and then selected a large number of sequences belonging to the genotypes that were associated with the Senegalese sequences and maximum number of 2 sequences (*n* = 2) for remaining genotypes. As a result, we obtained non-redundant representative sequences (*n* = 89), which were named dataset 2.

To trace back the origin and dissemination of the virus in Senegal (West Africa), we constructed a resampled dataset by including all the sequences found within the same cluster(s) as the Senegalese sequences. This dataset incorporated all available full-genome DENV−3 sequences from West Africa. This process led to the creation of dataset 3, which comprised DENV−3 sequences from Senegal as well as 43 sampled strains from various parts of the world.

The different datasets were aligned using MAFFT v7.490 software [22]. We then conducted screening for recombinant sequences using all the algorithms available in the RDP4 program, including RDP, GENECONV, BootScan, MaxChi, Chimaera, SiScan, and 3 Seq, all with the standard default parameters [23]. The sequence alignments, free of recombination, were carefully reviewed and edited manually using the AliView v.1.18 program [24].

Sequences of vaccine strains CYD-3 and TV003 were combined with Senegalese sequences, trimmed to the CprM-E gene region, and translated into amino acids using the AliView v.1.18 program [24] to construct dataset 4. Changes compared to the 5J7 B-cell mAB epitope were retrieved from dataset 5, which contained the amino acid sequences from the Senegalese DENV−3 sequences and a mAB epitope protein sequence.

To include as many Senegalese sequences as possible, population genetics and PCA analysis were performed on full E-gene sequences and organized as dataset 6 (*n* = 48).

### 2.7. Phylogenetic Analysis

Viral phylogenies based on nearly complete genomes and E-gene sequences for dataset 2 and dataset 3 were estimated using the maximum likelihood (ML) phylogenetic approach implemented in IQ-TREE v.1.5.5 software [25], and ModelFinder was used to set up the best fitting model according to the Bayesian information criterion (BIC) value [26]. Tree topology robustness was tested using 1000 replicates for the bootstrap value. Generated trees were visualized using FigTree v.1.4.3 (http://tree.bio.ed.ac.uk).

### 2.8. SNP Calling and Population Genetics Analysis

In dataset 6, single nucleotides polymorphisms (SNPs) were called and used to plot principal component analysis using R packages adegenet [27] and ape [28]. On the same dataset, pairwise FsT and analysis of molecular variance (AMOVA) were computed using Arlequin software v3.5.2.2 [29] (Table 1). All figures were plotted using the ggplot package implemented in R software v 3.6.0 [30].

### 2.9. Bayesian Phylogeographic Analysis

Molecular clock and data quality of dataset 3 were assessed using TempEst v.1.5.3 [31]. The spatiotemporal spread of DENV−3 in Senegal was reconstructed under a Bayesian framework implemented in BEAST. v.1.10.4 [30].

We employed the general time-reversible model with a gamma-distributed rate variation substitution model (GTR + G), chosen based on the Akaike information criterion (AIC), as determined by jModelTest v.2.1.10.

In line with previous findings on the evolutionary dynamics of related DENV, we examined the fit of an uncorrelated relaxed molecular clock, assuming a lognormal distribution. Additionally, we tested three non-parametric population growth models: (i) the standard Bayesian skyline plot (BSP, using 10 groups), (ii) the Bayesian skyride plot, and (iii) the Bayesian skygrid model (see Table 2).

To estimate phylogeographical patterns and parameters, we ran the Markov chain Monte Carlo (MCMC) for 50 million states, sampling every 50,000 states, and discarding the initial 10% as burn-in. We assessed MCMC convergence by ensuring an effective sample size (ESS) of greater than 200 using Tracer v.1.7.1 [32].

The resulting maximum clade credibility (MCC) tree was visualized and edited using FigTree v.1.4.4 (http://tree.bio.ed.ac.uk). To calculate the log marginal likelihood for the molecular clock and demographic model selection, we employed path sampling (PS) and stepping stone (SS) sampling approaches, running 100 path steps, each consisting of 1 million iterations.

### 2.10. Amino Acid Changes against Vaccinal Strains and Human DENV−3 mAb Epitopes

Using dataset 4, the amino acid changes in contemporary Senegalese DENV−3 strains compared to the vaccine strains of the same serotype (Dengvaxia CYD-3 and Tetravax-DV-TV003) were generated using a script written in the Python language that produced a tab-separated file containing the position where the amino acid changes occurred and the corresponding alternative allele, depending on the reference chosen (Dengvaxia sequence (CYD-3) and Tetravax-DV-TV003).

For DENV−3 mAB analysis, dataset 5 was aligned to identify the amino acid changes against the known 5J7 mAb epitope; changes were called as described below. The amino acid changes were represented using R software v3.6.0 [30].

## 3. Results

### 3.1. Spatial and Temporal Distribution of Sequenced Strains

**Figure 2:**
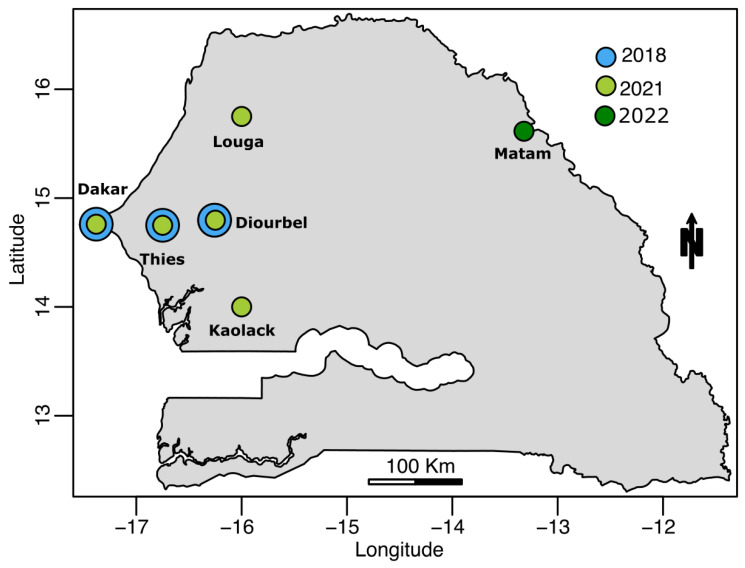
Map showing the spatial and temporal distributions of DENV−3 strains sequenced during this study. Dots indicate coordinates of sampling locations and colors indicate year (blue, 2018; light green, 2021; dark green, 2022).

During this work, we successfully sequenced 21 nearly complete genomes and at least complete CprM-E genes for 15 subjects, putting the overall obtained sequences at 36. According to the year of collection, seven of the sequences were sampled in 2018, nineteen in 2021, and ten in 2022. None of the sequenced strains were collected in 2019 or 2020. At the spatial level, the sequenced strains (*n* = 36) were collected around six localities, including Dakar (*n* = 7), Thies (*n* = 7), Diourbel (*n* = 3), Kaolack (*n* = 5), Louga (*n* = 2), and Matam (*n* = 12) (Cf Figure 2).

### 3.2. Phylogenetic Relationships of Senegalese DENV−3

**Figure 3:**
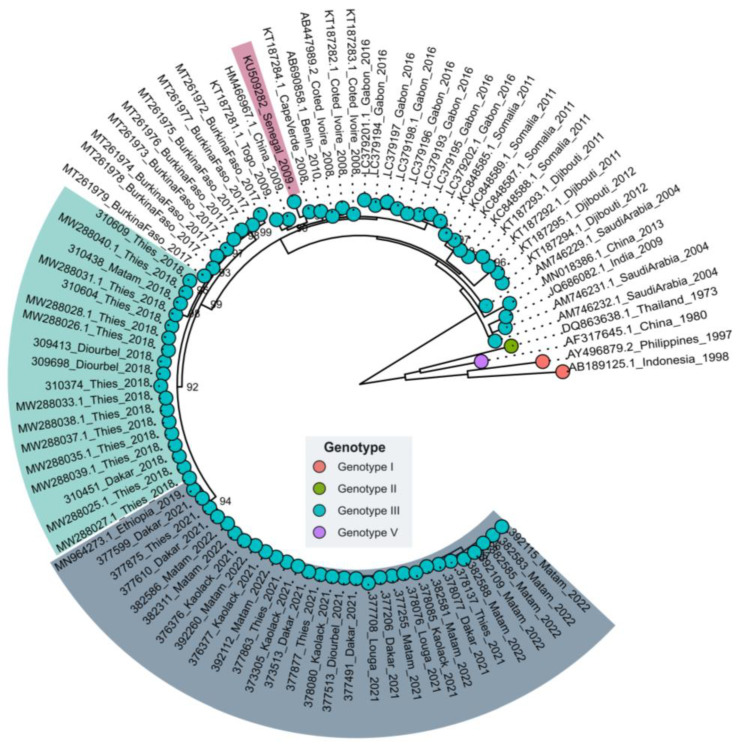
Maximum likelihood (ML) tree based on the E gene of DENV−3 sequences, showing the relationship with strains obtained in Africa and globally. The tree was drawn using IQ-tree [25], and TN + F + G4 model was used based on BIC criterion; 1000 replicates were used for robustness. Bootstrap values greater than 70 are shown on the tree. Tips are colored according to genotype, as described in the legend. Clusters of Senegalese strains sampled in 2009, 2018, and 2022 are highlighted in red, green, and blue, respectively.

Phylogenetic analysis classified the Senegalese DENV−3 sequences into genotype III (Figure 3). Interestingly it showed that they clustered in three separate clades within this particular genotype. Sequences of Senegalese strains sampled in 2018 clustered with isolates from Burkina Faso in 2017 (MT261972–MT261978). Those sampled in 2009 clustered with strains from Asia (China, 2009) and West African countries such as Benin, Cabo Verde, and Cote d’Ivoire between 2008 and 2009. Finally, sequences of strains collected in 2021/2022 were closely related to a unique strain sequenced in China in 2019 from a traveler who had returned from Ethiopia (MN964273.1). The same clustering pattern was found with nearly complete genomes despite the scarcity of full-genome sequences within Africa (Figure S1).

**Figure 4:**
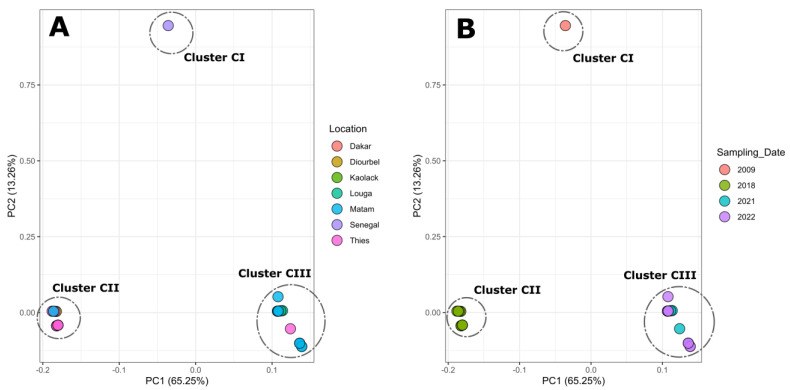
Principal component analysis (PCA) of Senegalese DENV−3 strains based on E-gene SNPs. Clustering shows that Senegalese DENV−3 strains clustered according to the year of sampling (**B**) instead of the location of sample provenance (**A**).

Principal Component Analysis (PCA) shows that Senegalese’s DENV−3 sequences analysis during this work clusters in different groups according to the year of sampling (Figure 4A) instead of the locations of sampling (Figure 4B).

**Figure 5:**
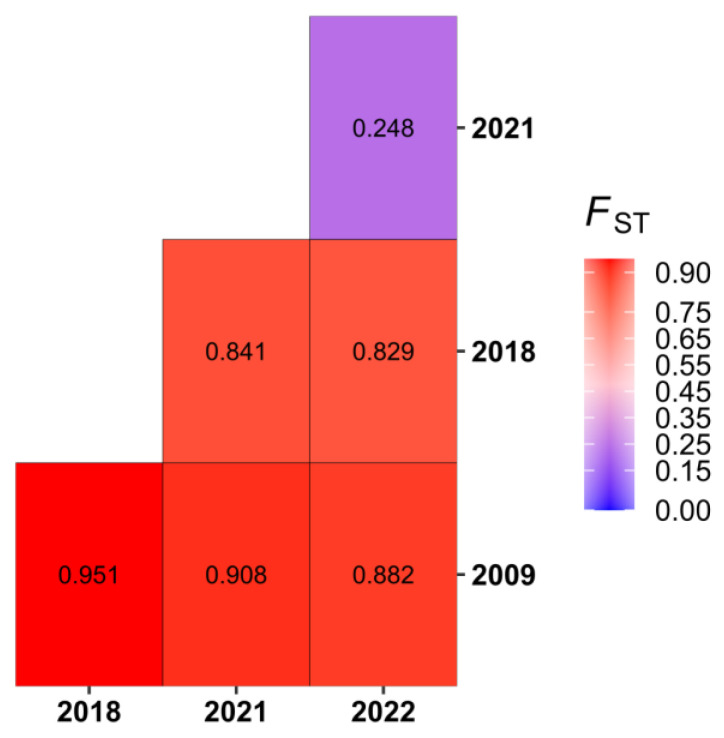
Pairwise genetic differentiation factor (F_ST_) between the population of DENV−3 sequences obtained during this study. Here, samples from the same year are considered to belong to the same population.

The determination of the genetic differentiation factor (FST) revealed a strong genetic differentiation among the studied populations, with *p* values < 0.05, except for the comparison between the SN_2021 and SN_2022 populations (*p* = 0.12613) (Figure 5; Table S1). However, the AMOVA test showed that over 80% of the genetic variability was interpopulation (Table 1).

### 3.3. Phylogeographic Pattern of DENV−3 Circulation in Senegal

**Figure 6:**
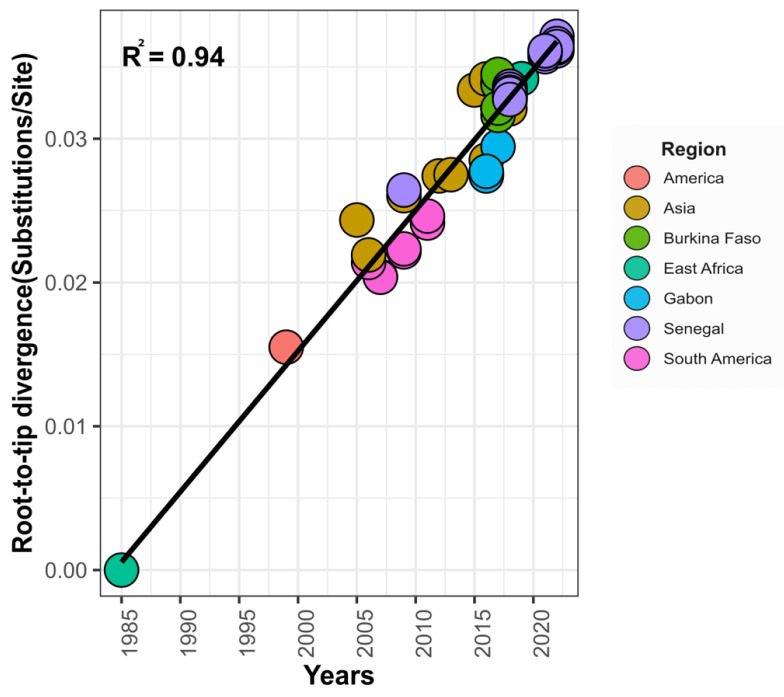
A regression of root-to-tip genetic distance against the time of sampling and showing a positive relationship (r = 0.97 and r^2^ = 0.94) indicative of a high rate of evolutionary change over the sampling period. Root-to-tip distances were calculated using a ML phylogeny using dataset 2.

The spatiotemporal spread of the DENV−3 strains sampled in Senegal between 2009 and 2022 was reconstructed and contextualized in a global movement during this study. A root-to-tip analysis showed that the virus accumulated genetic diversity over time (r = 0.97) (Figure 6).

Comparison of the best fitting model for virus propagation and diffusion to maximize viral dispersion pattern accuracy showed that the Skygrid demographic growth model was the best based on the path sampling and stepping stone methods (Table 2).

Phylogeographic analysis based on the nearly complete genome sequences showed that the Senegalese strains originated from Asia and West Africa (Burkina Faso) and were introduced in Senegal in three independent time periods between 2009 and 2022. The unique Senegalese sequence sampled in 2009 from a traveler who had returned to Italy was introduced into Senegal (location probability = 0.47) from Asia in 2005.64 (95% HPD: 2004.22–2007.06).

**Figure 7:**
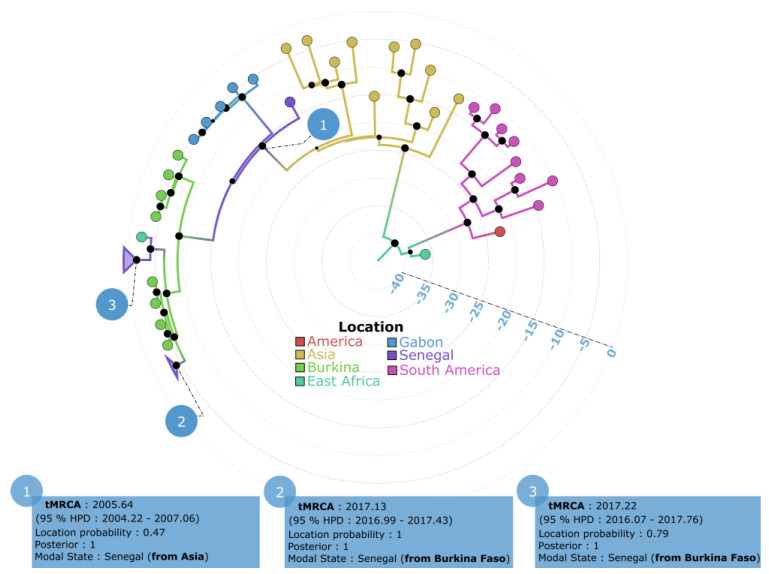
Bayesian discrete phylogeography based on nearly complete genome of DENV−3 strains isolated in Senegal between 2009 and 2022. Senegalese strains are grouped into three separate clusters. Information related to each cluster is indicated in the blue boxes, including tMRCA and origin. The black dots on the nodes represent the posterior probability value, and their size is proportional to their value. Each tip is colored according to the location of sampling of the corresponding sequence.

The two other recent clades in Senegal (Senegal 2018 and Senegal 2021/2022) had tMRCAs of 2017.1 (95% HPD: 2016.99–2017.43) and 2017.22 (95% HPD: 2016.07–2017.76), respectively. The strains belonging to these two clades were all probably introduced from Burkina Faso, with respective location probabilities of 1 and 0.79 (Figure 7).

### 3.4. In Silico Assessment of Impact of Amino Acid Changes against the Vaccines (CYD-3 and TV003) and Human mAB (mAB 5J7)

**Figure 8:**
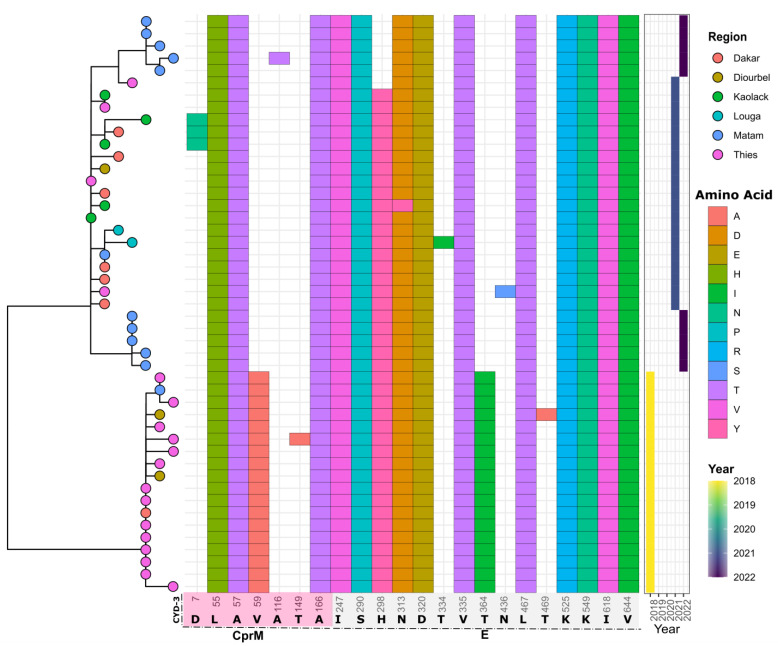
Mapping of observed amino acid changes among Senegalese DENV−3 strains when compared to the CYD-3 vaccine. The figure contains three panels: phylogenetic tree showing the relationship of Senegalese DENV−3 sequences (**left**), observed amino acid changes among Senegalese DENV−3 strains in comparison to the CYD-3 vaccine (**middle**), and year the sample was sequenced (**right**). Only the positions where amino acid changes were observed were plotted, with the amino acid position indicated below the figure.

**Figure 9:**
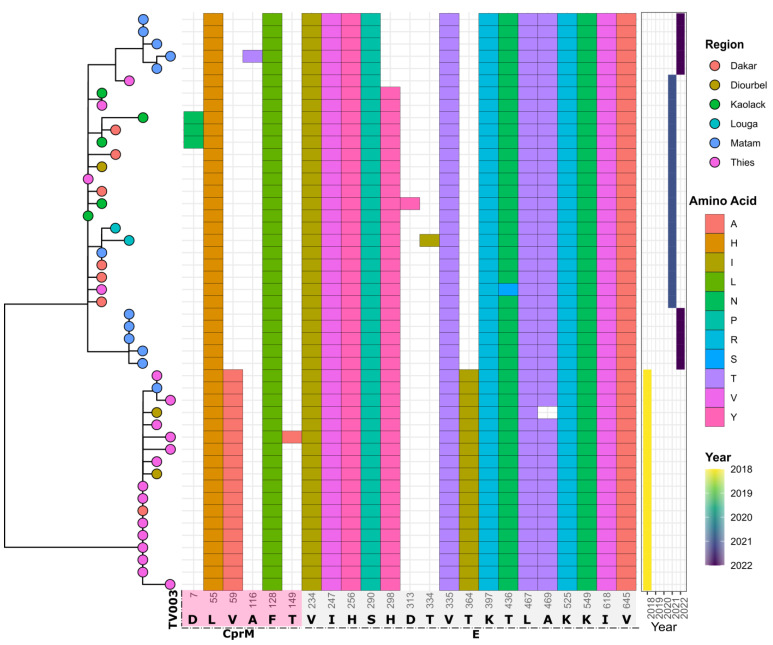
Mapping of observed amino acid changes among Senegalese DENV−3 strains when compared to the TV003 vaccine. The figure contains three panels: phylogenetic tree showing the relationship of Senegalese DENV−3 sequences (**left**), observed amino acid changes among the Senegalese DENV−3 strains in comparison to the TV003 vaccine (**middle**), and year of sample collection (**right**). Only the positions where amino acid changes were observed were plotted, with the amino acid position indicated below the figure.

The analysis showed that strains sequenced during this study and those from Senegal that were previously available in Genbank and sampled between 2018 and 2022 exhibited 22 substitutions when compared to the CYD-3 sequence. Amino changes were observed in the CprM gene (*n* = 7) and the E gene (*n* = 15). Compared to TV003, 23 substitutions were observed (*n* = 6 in the CprM and *n* = 17 in the E gene) (Figure 8 and Figure 9).

**Figure 10:**
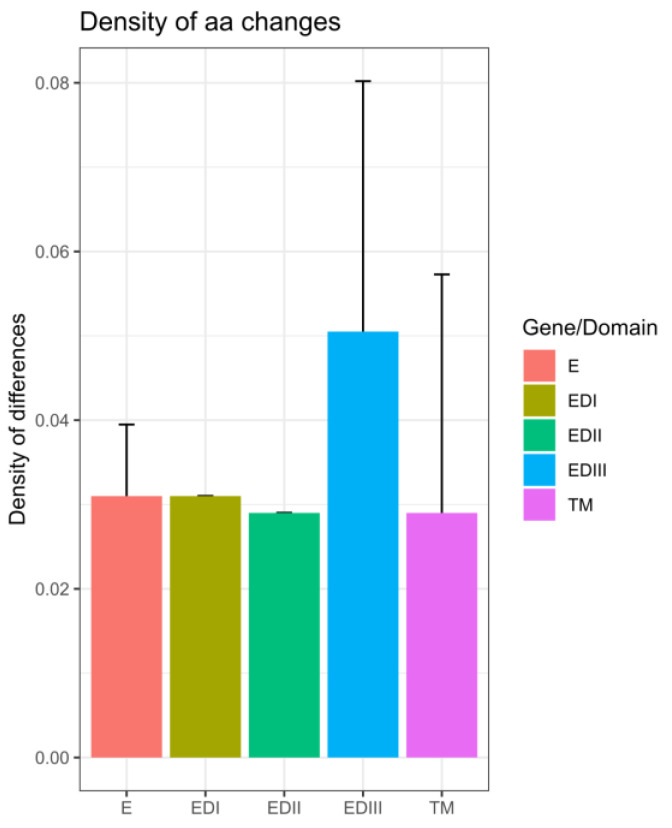
The variation in density (quantified as the number of differences relative to the domain’s length) is illustrated across distinct regions of the envelope protein, including envelope domains EDI-III and the transmembrane (TM) region. The accompanying error bars depict the standard deviation observed across the two vaccine strains.

Within the E gene, most of the changes between the sequenced Senegalese strains and vaccine strains were located in domain III (ED III) (Figure 10).

**Figure 11:**
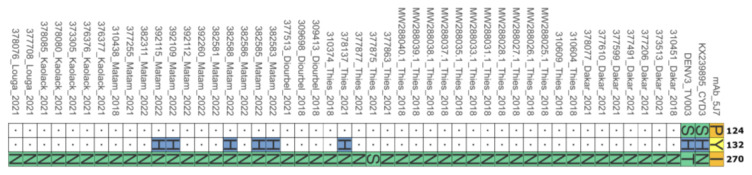
Observed amino acid changes when comparing contemporary DENV−3 strains with the known 5J7 B-cell epitope. Only positions where changes were observed are indicated on this figure. The numbers on the right refer to the amino acid position.

When comparing the sequences from Senegal and the vaccine strains to the B-cell epitope of DENV−3, there was a disparity at three sites (124, 132, and 270). Two positions (132 and 270) among Senegalese sequences showed amino acid changes when compared to mAb 5J7 B-cell epitopes (Figure 11).

## 4. Discussion

The first noticed urban dengue epidemic in Senegal was reported in 2009 and was caused by DENV−3 [33]. At the same time, many other West African countries reported epidemics caused by the same serotype [8,9]. In Senegal, the DENV−3 serotype re-emerged in 2018 in the context of a large religious mass gathering event in the Touba area [34]. After the event, DENV−3 cases increased and spread over the country, as exemplified by the reported epidemic in Agnam Civol (unpublished data). Despite the subregional notifications of DENV−3 circulation in Cape Verde, Cote d’Ivoire, Gabon, Burkina Faso, and Senegal (Thies, Touba) [9,35], genomic data about this serotype are limited [16]. Our work describes results from the first nationwide study leveraging nanopore sequencing to study the molecular evolution of DENV−3 in Senegal from 2009 to 2022. Our amplicon-based approach allowed us to retrieve 21 nearly complete genomes and 15 partial sequences for which at least a complete CprM and E gene were obtained. At the spatial level, sequenced DENV−3 strains were collected from six administrative regions of Senegal, namely Dakar, Thies, Diourbel, Louga, Kaolack, and Matam, highlighting the wide distribution of the serotype, as previously reported by Dieng and colleagues [11]. In Africa, this serotype was first reported in Mozambique in 1985 [36]. Since then, DENV−3 has been known to be endemic and actively circulating in West Africa since 2006 [9]. The current phylogenetic analysis showed that all Senegalese DENV−3 sequences belonged to genotype III in a cluster comprising strains isolated in numerous African countries, such as Gabon, Burkina Faso, Togo, Benin, and Côte d’Ivoire. These findings corroborate previous work which shows that most African DENV−3 belongs to genotype III, which is the main circulating genotype in the continent [7]. Interestingly, the phylogenetic tree showed that the Senegalese DENV−3 sequences analyzed during this work clustered in three different clusters with respect to the year of sampling (Figure 2). The results were corroborated by conducting SNP analysis through principal component analysis (PCA), as illustrated in Figure 4. This analysis indicated that the majority of the DENV−3 genomes from Senegal can be categorized into three distinct groups based on the year they were sampled rather than their geographic collection locations.

Interestingly, AMOVA analysis showed a strong genetic differentiation in the F_ST_ values, according to the year of collection (Figure 5; Table 1). This highlights an intense inter-location exchange of strains, thanks to travel, trade, and economic activities between regions [37].

The phylogeographic analysis (Figure 6; Table 2; Figure 7) confirmed at least three independent introduction events of DENV−3 in Senegal between 2009 and 2022. The first introduction event was from Asia in 2005.64 (95% HPD: 2004.22–2007.06). This strain occupies a basal position among the full-genome sequences obtained from Africa and probably spread, leading to the lineage that circulated in Gabon and Burkina Faso in 2016 and 2017, respectively (Figure 7). Given the availability of only one complete DENV−3 genome sequence from 2009 in the GenBank database, this estimation lacks robustness. Nonetheless, the results support earlier studies indicating that African DENV−3 infections were caused by viruses originating from the Indian subcontinent [38].

The recent clades in Senegal (Senegal, 2018, and Senegal, 2021/2022) had tMRCA estimates of 2017.1 (95% HPD: 2016.99–2017.43) and 2017.22 (95% HPD: 2016.07–2017.76), respectively. These estimations align with those of Dieng et al. in January 2017, which revealed the introduction of DENV−3 in Touba with a tMRCA of 2017.07 (95% HPD: 2016.61–2017.57) [12]. Strains belonging to these two clades were all likely introduced from Burkina Faso (with probabilities of origin being 1 and 0.79, respectively). A genomic study on a DENV outbreak in Thies, Senegal, in 2018 showed that the DENV−3 strains were closely related to those from the Gabonese outbreak in 2016–2017 [13]. Compared to the Thies study, our work updates the introduction routes of DENV−3 in Senegal by adding new strains from Senegal and including strains from Burkina Faso that were not available during the Thies study. Notably, Gaye et al.’s tMRCA estimate was based on sequences from Thies in 2018 and closely related African sequences from a Gabonese outbreak in 2016–2017, spanning about 13 years. This indicates a lack of recent evolutionary history, likely due to the limited use of only seven complete African genomic sequences at that time and the absence of DENV−3 sequences from Burkina Faso, 2017 and Senegal, 2018 (Touba), 2021, and 2022. With the inclusion of DENV−3 sequences from Burkina Faso, 2017 and the newly obtained sequences in our study, we have refined the resolution, providing a tMRCA for recent Senegalese DENV−3 sequences from around 4 years ago, aligned with the closely related Burkina Faso sequences from 2017.

From these results, we can infer the endemicity of DENV−3 that is associated with broad distribution in West Africa, as evidenced by previous studies [9,13]. Vaccine development against dengue has progressed during this decade [39]. However, the genetic diversity of DENV poses a major challenge to obtaining an effective vaccine [40]. Analysis of amino acid changes compared to vaccine strains showed twenty-two and twenty-three substitutions in relation to the CYD-3 and TV003 vaccines, respectively (Figure 8 and Figure 9). These amino acid changes could have a significant impact on the sensitivity and efficacy of these vaccines. Among factors influencing vaccine efficacy is the mismatch between vaccines and circulating strains [41]. Compared to a similar study in Burkina Faso based on the 2017 epidemic and global strains, Senegal’s DENV−3 strains exhibit their own genomic diversity, potentially compromising the efficacy of a DENV vaccine against this serotype in Senegal. These results emphasize the need for whole-genome sequencing of contemporary DENV strains circulating in Africa to update the virus’s antigenic evolution. Just as influenza viruses undergo antigenic evolution requiring frequent updates to vaccine antigens [42], the approach we used in our study, based on nanopore sequencing, could enhance the cost-effective genomic surveillance of DENV in Africa. Most of the observed amino acid changes against the vaccine strains were located in the E-gene domain III (ED III) (Figure 11), which is part of the exposed part of the E protein [43] and is known to enable long-lasting protective immunity against dengue virus infection [44]. This domain is known to play an important role in defining viral antigenicity [45]. Additionally, studies show that this region is the primary target of neutralizing antibodies [46,47]. According to this finding, in-depth characterizations of the observed changes in the ED III domain on antibody affinity are urgently needed [43] to assess the efficacy of prospective dengue vaccines in Africa.

Additionally, comparison of Senegal’s sequences and vaccine strains with DENV−3 B-cell epitopes reveals strong similarities for known B-cell epitope targets but divergence at three sites (124, 132, and 270) (Figure 11). In contrast to an earlier Burkina Faso study [16] where only one mutation at position 124 was observed, our study revealed two additional mismatches in Senegal’s DEN-3 sequences. These amino acid changes could disrupt the binding between the envelope protein and the heavy chain of monoclonal antibodies. Indeed, a single residue change through mutation or post-translational modification can alter epitope recognition [48]. These mutations could enhance pathogenicity by preventing binding with neutralizing monoclonal antibodies [48]. Given the identified positions, more comprehensive studies should investigate the role of the observed amino acid changes in vaccine efficacy and better characterize the immune response following DENV−3 infection.

Our work adds further evidence of intra-African DENV genetic variability, as previously raised by Letizia and colleagues [16]. For future vaccination campaigns against DENV in Africa, we should consider candidates which take into account contemporary genetic and antigenic variability within the African continent [16].

## 5. Conclusions

This work represents the first multiyear, country-wide study on the genetic diversity of DENV−3 in Senegal and the impact of observed diversity on prevention countermeasures since the first notification of this serotype in 2009. The study leveraged nanopore sequencing to characterize full-genome sequences of DENV in Africa, where genomic data are known to be limited. The protocol that was developed and validated in this work showed reliability and usefulness and should be expanded to other African countries to allow timely assessment of DENV genomic epidemiology within the continent and deep knowledge of the genetic diversity of contemporary strains, which is key prior to any large-scale vaccination implementation.

## Figures and Tables

**Table 1:** Results of AMOVA analysis.

Source of Variation	Sum of Squares	Variance Components	Percentage Variation
Among populations	130.12	4.02	80.25
Within populations	43.46	0.99	19.74
Total	173.79	5.01	

**Table 2:** Model comparison of strict molecular clock and demographic growth models through path sampling (PS) and stepping stone (SS) methods. Bold numbers indicate the best fitting model.

Demographic Growth Model	Relaxed Molecular Clock	
	PS	SS
Skyride	−27,752.10	−27,752.40
**Skygrid**	**−27,732.34**	**−27,732.50**
Skyline	−27,737.07	−27,736.62

**PS**: path sampling; **SS**: stepping stone. The best fitting model is highlighted in bold.

## Data Availability

The data that support the findings of this study are available from the corresponding author upon reasonable request.

## Supplementary Materials

The following are available online at https://www.mdpi.com/article/10.3390/vaccines11101537/s1: Table S1: Pairwise FST between sequences groups and associated *p*-values; Figure S1: Maximum likelihood (ML) tree based on nearly complete genome of Senegalese DENV−3 sequences, showing relationship with strains obtained in Africa and globally. The tree was drawn using IQ-tree [25], and TIM2 + F + G4 model was used based on BIC criterion; 1000 replicates were used for robustness. Bootstrap values greater than 70 are shown on the tree.

## References

[1] Harapan H., Michie A., Sasmono R.T., Imrie A. (2020). Dengue: A Minireview. Viruses.

[2] Chambers T.J., Hahn C.S., Galler R., Rice C.M. (1990). Flavivirus Genome Organization, Expression, and Replication. Annu. Rev. Microbiol..

[3] Katzelnick L.C., Fonville J.M., Gromowski G.D., Arriaga J.B., Green A., James S.L., Smith D.J. (2015). Dengue viruses cluster antigenically but not as discrete serotypes. Science.

[4] Lim J.K., Carabali M., Lee J.S., Lee K.S., Namkung S., Lim S.K., Yoon I.K. (2018). Evaluating dengue burden in Africa in passive fever surveillance and seroprevalence studies: Protocol of field studies of the Dengue Vaccine Initiative. BMJ Open.

[5] Bhatt S., Gething P.W., Brady O.J., Messina J.P., Farlow A.W., Moyes C.L., Hay S.I. (2013). The global distribution and burden of dengue. Nature.

[6] Amarasinghe A., Kuritsky J.N., Letson G.W., Margolis H.S. (2011). Dengue Virus Infection in Africa. Emerg. Infect. Dis..

[7] Ayolabi C.I., Olusola B.A., Ibemgbo S.A., Okonkwo G.O. (2019). Detection of Dengue viruses among febrile patients in Lagos, Nigeria and phylogenetics of circulating Dengue serotypes in Africa. Infect. Genet. Evol..

[8] Franco L., Di Caro A., Carletti F., Vapalahti O., Renaudat C., Zeller H., Tenorio A. (2010). Recent expansion of dengue virus serotype 3 in West Africa. Eurosurveillance.

[9] Abe H., Ushijima Y., Loembe M.M., Bikangui R., Nguema-Ondo G., Mpingabo P.I., Yasuda J. (2020). Re-emergence of dengue virus serotype 3 infections in Gabon in 2016–2017, and evidence for the risk of repeated dengue virus infections. Int. J. Infect. Dis..

[10] Abreu C., Silva-Pinto A., Lazzara D., Sobrinho-Simões J., Guimarães J.T., Sarmento A. (2016). Imported dengue from 2013 Angola outbreak: Not just serotype 1 was detected. J. Clin. Virol..

[11] Dieng I., Ndione MH D., Fall C., Diagne M.M., Diop M., Gaye A., Faye O. (2021). Multifoci and multiserotypes circulation of dengue virus in Senegal between 2017 and 2018. BMC Infect. Dis..

[12] Dieng I., Fall C., Barry M.A., Gaye A., Dia N., Ndione M.H.D., Sall A.A. (2022). Re-Emergence of Dengue Serotype 3 in the Context of a Large Religious Gathering Event in Touba, Senegal. Int. J. Environ. Res. Public Health.

[13] Gaye A., Ndiaye T., Sy M., Deme A.B., Thiaw A.B., Sene A., Ndiaye D. (2021). Genomic investigation of a dengue virus outbreak in Thiès, Senegal, in 2018. Sci. Rep..

[14] Torres-Flores J.M., Reyes-Sandoval A., Salazar M.I. (2022). Dengue Vaccines: An Update. BioDrugs.

[15] Usme-Ciro J.A., Méndez J.A., Laiton K.D., Páez A. (2014). The relevance of dengue virus genotypes surveillance at country level before vaccine approval. Hum. Vaccines Immunother..

[16] Letizia A.G., Pratt C.B., Wiley M.R., Fox A.T., Mosore M., Agbodzi B., Sangaré L. (2022). Retrospective Genomic Characterization of a 2017 Dengue Virus Outbreak, Burkina Faso. Emerg. Infect. Dis..

[17] Hill S.C., de Vasconcelos J.N., Granja B.G., Thézé J., Jandondo D., Neto Z., Afonso J.M. (2019). Early Genomic Detection of Cosmopolitan Genotype of Dengue Virus Serotype 2, Angola, 2018. Emerg. Infect. Dis..

[18] Dieng I., Barry M.A., Talla C., Sow B., Faye O., Diagne M.M., Faye O. (2022). Analysis of a Dengue Virus Outbreak in Rosso, Senegal 2021. Trop. Med. Infect. Dis..

[19] Wagner D., de With K., Huzly D., Hufert F., Weidmann M., Breisinger S., Bauer T.M. (2004). Nosocomial Acquisition of Dengue. Emerg. Infect. Dis..

[20] Dieng I., Talla C., Fauver J., Ndiaye M., Sagne S.N., Barry M.A., Faye O., Aparecida Sperança M. (2023). Reemergence of Sylvatic Dengue Virus in Southern Senegal, 2021. Infectious Diseases.

[21] Dieng I., Diallo A., Ndiaye M., Mhamadi M., Diagne M.M., Sankhe S., Faye O. (2022). Full genome analysis of circulating DENV-2 in Senegal reveals a regional diversification into separate clades. J. Med. Virol..

[22] Katoh K., Misawa K., Kuma K.I., Miyata T. (2002). MAFFT: A novel method for rapid multiple sequence alignment based on fast Fourier transform. Nucleic Acids Res..

[23] Martin D.P., Murrell B., Golden M., Khoosal A., Muhire B. (2015). RDP4: Detection and analysis of recombination patterns in virus genomes. Virus Evol..

[24] Larsson A. (2014). AliView: A fast and lightweight alignment viewer and editor for large datasets. Bioinformatics.

[25] Nguyen L.T., Schmidt H.A., von Haeseler A., Minh B.Q. (2015). IQ-TREE: A Fast and Effective Stochastic Algorithm for Estimating Maximum-Likelihood Phylogenies. Mol. Biol. Evol..

[26] Kalyaanamoorthy S., Minh B.Q., Wong T.K.F., von Haeseler A., Jermiin L.S. (2017). ModelFinder: Fast model selection for accurate phylogenetic estimates. Nat. Methods.

[27] Jombart T. (2008). *adegenet*: A R package for the multivariate analysis of genetic markers. Bioinformatics.

[28] Paradis E., Claude J., Strimmer K. (2004). APE: Analyses of Phylogenetics and Evolution in R language. Bioinformatics.

[29] Excoffier L., Laval G., Schneider S. (2007). Arlequin (version 3.0): An integrated software package for population genetics data analysis. Evol. Bioinform..

[30] R Core Team (2021). R Core Team. R: A Language and Environment for Statistical Computing.

[31] Rambaut A., Lam T.T., Max Carvalho L., Pybus O.G. (2016). Exploring the temporal structure of heterochronous sequences using TempEst (formerly Path-O-Gen). Virus Evol..

[32] Rambaut A., Drummond A.J., Xie D., Baele G., Suchard M.A. (2018). Posterior Summarization in Bayesian Phylogenetics Using Tracer 1.7. Syst. Biol..

[33] Faye O., Ba Y., Faye O., Talla C., Diallo D., Chen R., Sall A.A. (2014). Urban Epidemic of Dengue Virus Serotype 3 Infection, Senegal, 2009. Emerg. Infect. Dis..

[34] Sokhna C., Mboup B.M., Sow P.G., Camara G., Dieng M., Sylla M., Gautret P. (2017). Communicable and non-communicable disease risks at the Grand Magal of Touba: The largest mass gathering in Senegal. Travel Med. Infect. Dis..

[35] Tarnagda Z., Cissé A., Bicaba B.W., Diagbouga S., Sagna T., Ilboudo A.K., Muscatello D.J. (2018). Dengue Fever in Burkina Faso, 2016. Emerg. Infect. Dis..

[36] Gubler D.J. (1998). Dengue and Dengue Hemorrhagic Fever. Clin. Microbiol. Rev..

[37] Brunette G.W., Nemhauser J.B. (2019). Travel-Related Infectious Diseases. CDC Yellow Book 2020.

[38] Messer W.B., Gubler D.J., Harris E., Sivananthan K., De Silva A.M. (2003). Emergence and Global Spread of a Dengue Serotype 3, Subtype III Virus. Emerg. Infect. Dis..

[39] Khetarpal N., Khanna I. (2016). Dengue Fever: Causes, Complications, and Vaccine Strategies. J. Immunol. Res..

[40] Izmirly A.M., Alturki S.O., Alturki S.O., Connors J., Haddad E.K. (2020). Challenges in Dengue Vaccines Development: Pre-existing Infections and Cross-Reactivity. Front. Immunol..

[41] Gaillat J. (2019). Vaccin Grippe: L’efficacité en Question. https://www.sf2h.net/k-stock/data/uploads/2018/09/06062019_SESSION_PARALLELE_SP09_1640_Salle_Marie_Curie_1_Niveau_1_Jacques_GAILLAT.pdf.

[42] Kim H., Webster R.G., Webby R.J. (2018). Influenza Virus: Dealing with a Drifting and Shifting Pathogen. Viral Immunol..

[43] Jagtap S., Pattabiraman C., Sankaradoss A., Krishna S., Roy R. (2023). Evolutionary dynamics of dengue virus in India. PLoS Pathog..

[44] Muné M., Rodríguez R., Ramírez R., Soto Y., Sierra B., Rodríguez Roche R., Guzmán M.G. (2003). Carboxy-terminally truncated Dengue 4 virus envelope glycoprotein expressed in Pichia pastoris induced neutralizing antibodies and resistance to Dengue 4 virus challenge in mice. Arch. Virol..

[45] Young E., Carnahan R.H., Andrade D.V., Kose N., Nargi R.S., Fritch E.J., Baric R.S. (2020). Identification of Dengue Virus Serotype 3 Specific Antigenic Sites Targeted by Neutralizing Human Antibodies. Cell Host Microbe.

[46] Chen S.P., Yu M., Jiang T., Deng Y.Q., Qin C.F., Han J.F., Qin E.D. (2008). Identification of a recombinant dengue virus type 1 with 3 recombination regions in natural populations in Guangdong province, China. Arch. Virol..

[47] Leng C.H., Liu S.J., Tsai J.P., Li Y.S., Chen M.Y., Liu H.H., Chen H.W. (2009). A novel dengue vaccine candidate that induces cross-neutralizing antibodies and memory immunity. Microbes Infect..

[48] Huang Y.J.S., Nuckols J.T., Horne K.M., Vanlandingham D., Lobigs M., Higgs S. (2014). Mutagenesis analysis of T380R mutation in the envelope protein of yellow fever virus. Virol. J..

