# Supplementary figures and images for: Molecular Evolution of Dengue Virus 3 in Senegal between 2009 and 2022: Dispersal Patterns and Implications for Prevention and Therapeutic Countermeasures
Source: Vaccines (Basel). 2023 Sep 28;11(10):1537. doi: 10.3390/vaccines11101537 (PMC10610876; doi:10.3390/vaccines11101537)

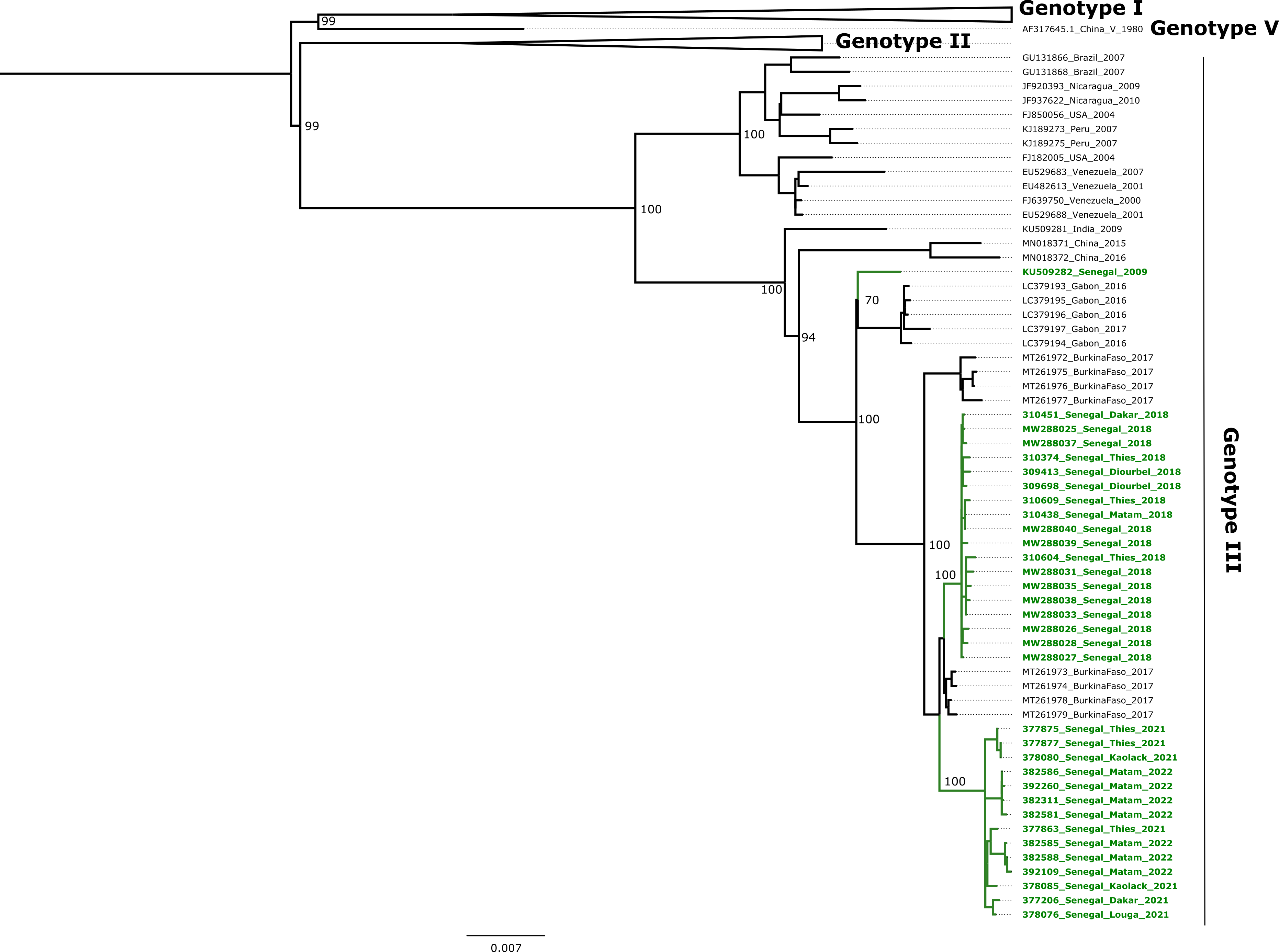

Supplement: Supplementary file 1 [file vaccines-11-01537-s001.zip › Figure S1.png]
